# Metabolomics Analysis Unveils the Underlying Mechanism of Low-Temperature Combined with Nitrogen Modified Atmosphere in Delaying Quality Deterioration of Rice

**DOI:** 10.3390/foods15132326

**Published:** 2026-07-01

**Authors:** Lulu Li, Yan Zhao, Yanan Zhao, Haoxin Lv, Wanxuan Huo

**Affiliations:** 1 School of Food and Strategic Reserves, Henan University of Technology, Zhengzhou 450001, China; lilulu1023@126.com (L.L.); zhaoyanan20182018@163.com (Y.Z.); lvhaoxin0129@126.com (H.L.); 2 School of International Education, Henan University of Technology, Zhengzhou 450001, China; 13835809348@163.com

**Keywords:** rice, low temperature, nitrogen modified, mycotoxins, nontargeted metabolomics

## Abstract

Rice storage under suboptimal conditions leads to rapid quality deterioration. In this study, effects of low-temperature (LT, 20 °C) combined with nitrogen-controlled atmospheric conditions (NCA, 95%) on physicochemical properties, nutrient, mycotoxins and metabolites of rice during 180 d storage were comprehensively evaluated, to explore a potential preservation technique for rice. The LT + NCA treatment significantly inhibited the accumulation in free fatty acid value, malondialdehyde and electrolyte leakage, while maintaining higher catalase activity, lower amylose content and better pasting properties (higher breakdown, lower final viscosity and setback). Crucially, mycotoxin accumulation remained within safe limits across all groups, with LT + NCA showing the lowest levels. Metabolomics analysis identified 653 metabolites, with LT + NCA significantly modulating pathways related to carbohydrate metabolism (e.g., promoting raffinose accumulation while inhibiting the pentose phosphate pathway) and amino acid metabolism (enhancing glutathione metabolism and suppressing arginine biosynthesis). These metabolic rearrangements reduced oxidative damage, stabilized membrane integrity, and preserved cooking quality. Therefore, LT + NCA could be a superior strategy for delaying oxidative stress, maintaining nutritional and cooking quality, and ensuring the safety of stored rice.

## 1. Introduction

Rice (*Oryza sativa* L.), a staple food for approximately 75% of the global population, faces increasing production demands driven by worldwide population growth [1,2,3]. According to FAO statistics in 2025, global rice consumption reached 537.2 million tons, with China being the largest producer. Given its seasonal production and constant consumption demand, storing rice under optimal conditions is essential to preserve its quality and safety over extended periods [4].

Beyond its caloric contribution, rice is a rich source of essential macronutrients, minerals, vitamins and bioactive phytochemicals [5]. However, the physicochemical properties and nutritional value of rice are highly susceptible to degradation during storage. Factors including temperature, gas composition, moisture content, relative humidity, and storage duration significantly influence its chemical composition and nutritional properties [6,7,8]. In addition, during storage, although paddy rice is dormant, ongoing physiological activities such as starch hydrolysis and lipid oxidation create a conducive environment for microbial invasion and toxin production [9]. Mycotoxin contamination in rice, primarily caused by storage fungi (Aspergillus, Penicillium, and Fusarium), is a critical postharvest issue that causes economic losses, quality deterioration and organoleptic spoilage, accounting for at least 10% of global stored cereal losses [8,10]. Therefore, it is important to adopt advanced preservation technologies and equipment and scientific storage technology for ensuring the long-term quality of stored rice.

Storage conditions significantly influence rice metabolic and physiological processes. Elevated temperatures and high oxygen concentrations accelerate lipid rancidity and quality deterioration [11,12]. In contrast, low-temperature storage suppresses respiration, harmful metabolic changes, fungal growth and pest incidence, thereby delaying aging and preserving quality [1]. Nitrogen-controlled atmosphere and reaeration storage can delay rice quality deterioration. A 96% N_2_ controlled atmosphere suppressed lipase and lipoxygenase activities, reducing lipid hydrolysis and oxidation, thus delaying deterioration [4]. Similarly, 90% N_2_ modified atmosphere packaging retards increase in fatty acid value and amylose content and slows the decline in enzyme activities [13].

In recent years, great attention has been devoted to low-temperature combined with modified atmosphere storage due to its unique impact across a wide range of industries and markets. Currently, this technology has been demonstrated to have a positive impact on food storage and preservation. For instance, Murmu & Mishra’s (2018) [14] study indicated that active modified atmosphere packaging with ethylene and moisture scavengers maintains quality of guava during low-temperature storage. Previous studies have shown that low temperature combined with nitrogen gas is highly effective in delaying the aging of high-moisture indica rice [15].

Despite these advancements, the metabolic profile and mycotoxin dynamics of safe moisture rice under low-temperature combined with nitrogen-controlled atmosphere (LT + NCA) storage remain insufficiently explored. This study therefore employs untargeted metabolomics to compare the effects of this combined condition on rice physiology, nutrient retention, metabolic changes, and mycotoxin content during 180 d of storage. This study aims to elucidate the molecular mechanisms by which LT + NCA storage delays deterioration and modulates postharvest metabolism, thereby providing a foundational dataset to evaluate its efficacy in extending the storage period of rice, minimizing undesirable physicochemical changes, and controlling mycotoxin contamination.

## 2. Materials and Methods

### 2.1. Materials

Rice of the variety “Huanghuazhan” was obtained from a local market in Wuhan (Hubei). Ethanol (95%, *v*/*v*), KOH and phenolphthalein were obtained from Tianjin Kemiou Chemical Reagent Co., Ltd. (Tianjin, China). Trichloroacetic acid (TCA) and thiobarbituric acid (TBA) were purchased from Shanghai Aladdin Biochemical Technology Co., Ltd. (Shanghai, China). Sodium hydroxide and acetic acid were supplied by Sigma-Aldrich Co. (St. Louis, MO, USA). All other chemicals were of analytical grades.

### 2.2. Rice Sample Preparation and Storage

All harvested rice were air dried after impurity removal, and the moisture content was adjusted to 13.5% according to the direct drying method specified in the Chinese national standard GB/T 5009.3-2016 (GAQS, 2016a) [16]. For each storage treatment, three independent biological replicate batches were prepared. Briefly, 2500 g of rice from each replicate batch was randomly distributed into a 0.24 mm polyethylene bag (40 cm × 35 cm) and placed in a constant temperature and humidity chamber (HWS-300, Ningbo Southeast Instrument Co., Ltd., Ningbo, China).

For conventional storage (CS), the samples were stored at 30 °C under atmospheric conditions. For low-temperature (LT) storage, the samples were stored at 20 °C under atmospheric conditions. For modified atmosphere storage, the rice samples were packaged under reduced pressure, using a modified atmosphere packaging machine to achieve a nitrogen concentration of 95%. The residual oxygen concentration in the package was measured weekly using a gas detector (MIC-600, Shenzhen Yiyuntian Technology Co., Ltd., Shenzhen, China) and maintained below 5.0 ± 0.1% during storage. The samples under nitrogen-controlled atmosphere (NCA) were stored at 30 °C, while those under low-temperature combined with nitrogen-controlled atmosphere (LT + NCA) were stored at 20 °C. All samples were stored for 180 d and collected at 30 d intervals for analysis. The collected samples were preserved at −80 °C until further analysis.

### 2.3. The Physiological Quality Changes in Rice During Storage

#### 2.3.1. The Fatty Acid Value

The fatty acid value (FAV) was determined in accordance with Qu et al.’s (2022) [13] method, with minor modifications. Briefly, 50 mL of anhydrous ethanol was added to 10.00 g of rice sample, shake well for 10 min and then filter. Then, 25 mL of filtrate was mixed with 50 mL of distilled water, and 3 drops of phenolphthalein indicator were added. The diluted filtrate was titrated with a standard potassium hydroxide solution. The FAV was expressed as the amount of potassium hydroxide required to neutralize the free fatty acids in 100 g of rice samples.

#### 2.3.2. Malondialdehyde Content

The malondialdehyde (MDA) content was measured according to the method of Gu et al. (2025) [17] with minor modifications. First, 1.0 g of rice was dissolved in 5.0 mL of 10% (*w*/*w*) trichloroacetic acid (TCA), ground and homogenized. The mixture was centrifuged at 4 °C and 10,000× *g* for 20 min to obtain the supernatant. Then, 2.0 mL of the supernatant was mixed with 2.0 mL of 0.67% thiobarbituric acid. For the control blank, 2.0 mL of 10% TCA solution was used instead of the extract. The mixture was heated in a boiling water bath for 20 min, cooled and centrifuged again. Finally, the absorbance of the supernatant was measured at 450 nm, 532 nm and 600 nm. The results were expressed as mg/kg.

#### 2.3.3. Catalase Activity

Catalase (CAT) activity in rice was determined using an enzyme-linked immunosorbent assay kit (Solarbio Sciences and Technology Co., Ltd., Beijing, China). The CAT activity was expressed as U/g.

#### 2.3.4. Detection of Relative Electrical Conductivity

Relative electrical conductivity was determined according to a previously published method [18], with slight modifications. Intact, plump and uniform brown rice were selected after husking. The kernels were rinsed three times with distilled water and placed in a beaker. Then, 50 mL of distilled water was added and the beaker was left to stand in a constant temperature box at 30 °C for 13 h. The conductivity was measured using a conductivity meter (DDS-307A, Shanghai Yi Electrical Scientific Instruments Co., Ltd., Shanghai, China), and the result was expressed as μs/cm.

#### 2.3.5. Color Determination

The color of rice was assessed using the method described by Zhao et al. (2024) [18]. A 10 g rice sample was placed in a color difference meter (CR-410, Konica Minolta, Tokyo, Japan) and the L* (brightness) and a* (redness-greenness) values were recorded. Each sample was measured in triplicate.

### 2.4. The Nutritional Quality Changes in Rice During Storage

#### 2.4.1. Amylose Content Measurement

Amylose content was conducted as described by Qu et al. (2022) [13]. Briefly, rice grains were ground into refined rice flour. The flour was then defatted by reflux extraction with petroleum ether for 6 h, followed by air-drying in a well-ventilated area for 24 h to allow complete evaporation of the petroleum ether. A 100 mg of aliquot of the defatted sample was placed into a 100 mL conical flask. Then, 1 mL of ethanol solution was carefully added to wet the sample and wash off any sample adhering to the flask wall. Subsequently, 9.0 mL of 1.0 mol/L sodium hydroxide solution was transferred into the flask, and the mixture was gently shaken. The mixture was heated in a boiling water bath for 10 min to disperse the starch. After heating, the flask was removed, cooled to room temperature, and the contents were transferred to a 100 mL volumetric flask. Distilled water was added to volume, and the solution was mixed well. An aliquot of 5.0 mL of the sample solution was accurately transferred into a 100 mL volumetric flask that had been pre-filled with approximately 50 mL of water. Then, 1.0 mL of acetic acid was added, the solution was shaken well, and then 2.0 mL of iodine reagent was added. After dilution to volume, the mixture was allowed to stand for 10 min, and the absorbance was measured at 720 nm. The standard curve was obtained as y = 0.0096x + 0.0246, with a correlation coefficient (R^2^) of 0.9987.

#### 2.4.2. Pasting Properties of Rice

The pasting viscosity of rice flour was measured using a rapid viscosity analyzer (RVA-TecMaster, Perten, Sweden) according to the method described by Gu et al. (2025) [19]. Briefly, 3.0 g of milled rice flour was added to simple cups containing 25 mL of distilled water. The pasting characteristics, including peak viscosity (PV), hot paste viscosity (HPV), final viscosity (FV), setback (SB), breakdown, and pasting temperature (PT), were recorded.

### 2.5. The Mycotoxins Content in Rice During Storage

The contents of aflatoxin B1(AFB1) and ochratoxin A (OTA) in rice were determined using commercial enzyme-linked immunosorbent assay (ELISA) kits (H010532B and SW-10100-07, Suwei Biological Research Co., Ltd., Wuxi, China). A standard curve was constructed by measuring the optical density (OD) of standard solutions (0, 0.1, 0.25, 0.5, 1.0 and 2.0 ng/mL) at 450 nm, and the AFB1 and OTA contents in rice were subsequently calculated from the curve obtained.

### 2.6. UHPLC-OE-MS Conditions for Metabolomics Analysis

#### 2.6.1. Metabolites Extraction

First, a 20 mg rice sample was taken at low temperature and mixed with 1000 μL of extraction solution (methanol: acetonitrile: deionized water, *v*/*v*/*v*) containing deuterated internal standards. The mixture was vortexed for 30 s, homogenized at 35 Hz for 4 min, and then sonicated in an ice water bath for 5 min. This sonication step was repeated 3 times. The mixture was then incubated at 40 °C for 1 h to precipitate proteins. After incubation, the sample was centrifuged at 13,800× *g* and 4 °C for 10 min. The supernatant was transferred to autosampler vials for analysis. The metabolomics analysis was conducted in 6 replicates for each sample. A quality control (QC) sample was prepared by mixing equal aliquots of all sample supernatants. During instrumental analysis, one QC sample was inserted every 5–15 samples to assess the repeatability of the entire analytical process.

#### 2.6.2. Non-Targeted Metabolomics Analysis

For non-polar metabolites, LC-MS/MS analysis was performed using a UPLC system (Vanquish, Thermo Fisher Scientific, Waltham, MA, USA) equipped with a Phenomenex Kinetex C18 column (2.1 mm × 50 mm, 2.6 μm). Mobile phase A was 0.01% acetic acid in water and mobile phase B was isopropanol: acetonitrile (1:1, *v*/*v*). The autosampler temperature was set to 4 °C, and the injection volume was 2 μL. The mass spectrometric data were collected using a Orbitrap Exploris 120 mass spectrometer (Orbitrap MS, Thermo Fisher Scientific) equipped with an electrospray ionization (ESI) source operating in positive mode and negative mode. The ESI source conditions were as follows: sheath gas flow rate 50 Arb, auxiliary gas flow rate 15 Arb, capillary temperature 320 °C, full MS resolution 60,000, MS/MS resolution 15,000, stepped normalized collision energy (SNCE) 20/30/40, and spray voltage 3.8 kV (positive) or −3.4 kV (negative).

#### 2.6.3. Data Preprocessing and Annotation

Raw data were converted to the mzXML format using ProteoWizard and processed with an in-house program developed in R based on XCMS for peak detection, extraction, alignment, and integration. Data preprocessing was performed as follows: metabolic features detected in at least 80% of any sample group were retained. Missing values were replaced with the minimum estimated level for each feature, and the data were normalized by total sum to correct for variations due to sample preparation and instrument instability. Variables with relative standard deviation (RSD) > 30% in quality control (QC) samples were excluded, and the remaining data were log10 logarithmicized to obtain the final matrix. The R package and the BiotreeDB (V3.0) were used for metabolite identification. All annotated metabolites were categorized following Metabolomics Standards Initiative (MSI) criteria: Level 1 (identified with authentic standards), Level 2 (putative annotation via database matching), Level 3 (only compound class determined), and Level 4 (unknown features). All differential metabolites in this study were classified as Level 1 or Level 2.

#### 2.6.4. Metabolomics Statistical Analysis

The data were subjected to multivariate data analyses, including principal component analysis (PCA) and orthogonal partial least squares-discriminant analysis (OPLS-DA). Sevenfold cross-validation and response permutation tests were used to evaluate the robustness of the models. The variable importance in projection (VIP) value of each variable in the OPLS-DA model was calculated to assess its contribution to classification. Metabolites with a VIP value > 1 were further subjected to Student’s *t*-test at the univariate level to determine significance. Identified metabolites were annotated using the KEGG Compound database (http://www.kegg.jp/kegg/compound/, accessed on 26 January 2026) and the KEGG Pathway database (http://www.kegg.jp/kegg/pathway.html, accessed on 18 February 2026). Pathways with significantly regulated metabolites were then subjected to metabolite set enrichment analysis (MSEA) and their significance was determined by the *p*-value of the hypergeometric test.

### 2.7. Statistical Analysis

All data were expressed as mean ± standard deviation (SD). Univariate analysis of variance (ANOVA) followed by Duncan’s multiple range test was performed using IBM SPSS 26.0 statistical software (IBM, New York, NY, USA). Differences were considered statistically significant at *p* < 0.05.

## 3. Results and Discussion

### 3.1. Effect of LT + NCA on FAV, MDA, Electrical Conductivity and CAT Activity of Rice During Storage

#### 3.1.1. FAV

**Figure 1:**
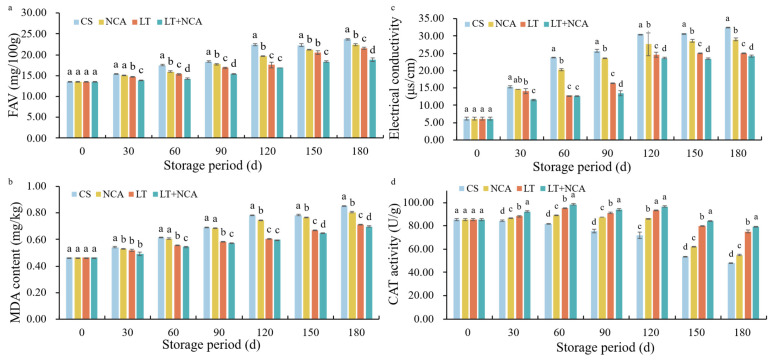
FAV (**a**), MDA (**b**), electrical conductivity (**c**) and CAT activity (**d**) of rice during 180 d of storage. CS, conventional storage; LT, low temperature; NCA, nitrogen-controlled atmospheric conditions, LT + NCA, low temperature combined with nitrogen-controlled atmospheric conditions. Results with different letters (a–d) in the same storage period are significantly different (*p* < 0.05).

FAV, reflecting the continuous hydrolysis and accumulation of lipids, was a key indicator of rice rancidity and quality [4,20,21]. As shown in Figure 1a, FAV increased with prolonged storage across all conditions (CS, NCA, LT and LT + NCA). FAV, an indicator of free fatty acid content in grain, is widely used to reflect the extent of lipid degradation and rice deterioration during storage [9,22,23]. The FAV of fresh rice was 13.54 mg/100 g, which increased to 23.71, 22.42, 21.53 and 18.84 mg/100 g after 180 d of storage under CS, NCA, LT and LT + NCA conditions, respectively, with the LT + NCA group showing the lowest increase. Notably, the FAV of rice stored under LT + NCA for 180 d (18.84 mg/100 g) was comparable to that of rice stored under CS for 90 d, indicating that LT + NCA storage significantly delayed FAV accumulation. This delaying effect can be attributed to the combined influence of low temperature and modified atmosphere. On the one hand, low temperature itself retards the increase in FAV, as reported by Qu et al. (2025) [21], who observed slower FAV growth at 15 and 20 °C. High storage temperatures typically accelerate lipid degradation and oxidative reactions [24]. On the other hand, modified atmosphere conditions such as high-nitrogen environments (e.g., 96% or 78% N_2_) also slow the rise in FAV by limiting oxygen availability, thereby suppressing lipid autoxidation, a primary mechanism of lipid deterioration during storage [4,11].

#### 3.1.2. MDA

**Figure 2:**
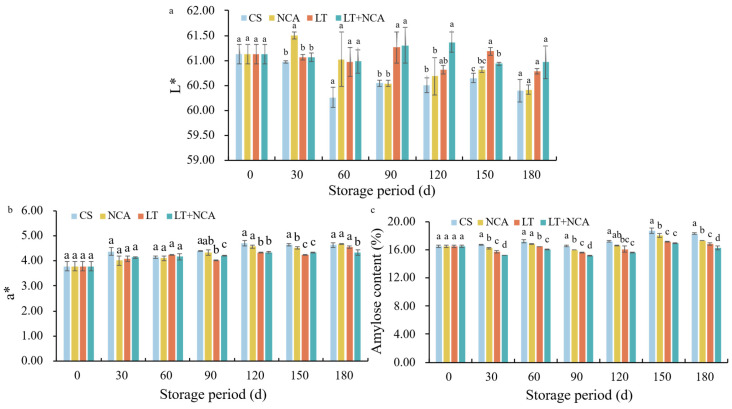
L* value (**a**), a* value (**b**) and amylose content (**c**) of rice during 180 d of storage. LT, low temperature; NCA, nitrogen-controlled atmospheric conditions, LT + NCA, low temperature combined with nitrogen-controlled atmospheric conditions. Results with different letters (a–d) in the same storage period are significantly different (*p* < 0.05).

MDA, a primary product of membrane lipid peroxidation, is a key biomarker of cellular senescence and membrane damage in stored rice [1,17]. As shown in Figure 2b, the MDA content of fresh rice was 0.4628 mg kg^−1^, increasing after 180 d to 0.8534 (CS), 0.8063 (NCA), 0.7121 (LT) and 0.6972 mg kg^−1^ (LT + NCA). For all samples, MDA content increased significantly over storage time, a trend consistent with the findings of L. Qu, et al. (2025) [21], indicating a gradual intensification of lipid peroxidation in rice. Notably, although MDA content continued to increase throughout storage under NCA, LT and LT + NCA conditions, it remained consistently lower than under CS conditions. This suggests that both LT and NCA help mitigate membrane damage by slowing MDA accumulation. Specifically, after 180 d, the lowest MDA content was observed under LT + NCA storage (0.6972 mg·kg^−1^). Compared with CS, reductions of 18.22%, 16.95% and 6.0% were observed under LT + NCA, LT and NCA treatments, respectively. The accumulation of MDA can attack the phospholipid bilayer, thereby damaging cell membrane structure [1]. These results demonstrate that LT combined with NCA most effectively suppresses MDA accumulation and alleviates oxidative damage in stored rice. These findings align with the existing literature. Liu et al. (2025) [1] reported lower MDA content and better quality in rice stored at low temperature (15 °C) compared with 25 °C, attributing this to reduced membrane lipid peroxidation at lower temperatures. Similarly, Wu et al. (2022) [25] found that modified atmosphere packaging (N_2_:O_2_ = 9:1) significantly lowered MDA accumulation in peanut kernels, likely due to reduced oxygen availability limiting oxidative damage during storage.

#### 3.1.3. Relative Electrical Conductivity

Electrolyte leakage rate is commonly used to assess membrane permeability in plants [25], as adverse storage conditions accelerate cell membrane peroxidation and electrical conductivity reflects the degree of membrane damage [26]. As shown in Figure 1c, the relative electrical conductivity (REC) of all rice samples increased with storage duration, indicating enhanced membrane permeability and loss of integrity. Specifically, after 180 d of storage, the REC increased by 4.34, 3.77, 3.13 and 2.99 times under CS, NCA, LT and LT + NCA treatments, respectively. Throughout the storage period, the REC of the LT + NCA group was significantly lower than that of the CS group (*p* < 0.05), indicating that LT + NCA condition effectively inhibited the increase in conductivity and helped maintain rice quality. After 180 d storage, the REC values of the NCA, LT and LT + NCA treatments were 10.76%, 22.66% and 25.40% lower, respectively, compared to the CS control. These results illustrated that NCA, LT and LT + NCA treatments can effectively delay the accumulation of REC, thereby mitigating cell membrane damage in rice. Zhang et al. (2026) [27] reported that low-temperature storage (1 °C) reduced the relative electrolyte leakage rate in peach fruit, thus best preserving membrane integrity. Wu et al. (2022) [25] observed that fresh edible peanut kernels treated with modified atmosphere packaging (N_2_:O_2_ = 9:1) exhibited relatively low electrical conductivity, likely due to reduced oxidative damage under low-oxygen conditions.

#### 3.1.4. Catalase

Catalase (CAT) is a crucial antioxidant enzyme that contributes to rice freshness by scavenging hydrogen peroxide generated during grain respiration [17]. Together with other enzymes like POD and SOD, CAT plays a vital role in protecting plant from oxidative damage [28]. As shown in Figure 1d, CAT activity in all samples exhibited a trend of initial increase followed by a decline throughout storage. The activity rose significantly during the early storage period (up to 60 d), which can be interpreted as an adaptive biochemical defense response to mounting oxidative stress. However, as storage progressed beyond this point, CAT activity began to decrease gradually. This decline likely reflects the eventual exhaustion of the rice’s defensive capacity, where overproduction of free radicals overwhelms and damages the enzymatic system, leading to irreversible loss of activity [29]. After 180 d of storage, CAT activity in rice under LT, NCA and LT + NCA treatment was significantly higher than that under CS conditions (*p* < 0.05). These results demonstrate that LT, NCA and LT + NCA storage conditions alleviate the decrease in CAT activity and mitigate oxidative damage, explaining the lower MDA content and electrolyte leakage observed in rice (Figure 1a,b). The finding is consistent with Gu et al. (2025) [17], who reported that low temperature enhances CAT activity and slows quality deterioration.

### 3.2. Changes in Color Measurement, Amylose Content and Pasting Properties of Rice During Storage

#### 3.2.1. Rice Color

Rice, as a biological material, is highly sensitive to storage temperature. Due to its physiological characteristics, fresh paddy with relatively low stress tolerance is prone to yellowing or browning when exposed to high-temperature and high-humidity conditions [8]. As shown in Figure 2a,b, the L* value (lightness) of rice decreased with prolonged storage time, indicating that the grains became darker. Concurrently, the a* value (redness/greenness) increased across all samples during storage. This rise in a* value is attributed to oxidation reactions involving fats and proteins within the rice. Furthermore, the abundant starch in rice grains can be converted to reducing sugars under certain conditions, which subsequently participate in Maillard reactions and generate reddish-brown compounds [30]. After 180 d of storage, the a* value of rice under the LT + NCA treatment was significantly lower than that under the CS, LT and NCA conditions (*p* < 0.05). These results indicated that the combined LT + NCA storage condition effectively inhibits the increase in a* value, thereby suppressing the oxidation reactions of fats and proteins and associated color deterioration of rice during storage.

#### 3.2.2. Amylose Content

Starch, comprising approximately 80% of rice grain mass, serves as its primary nutritional component. The edible quality of rice is affected by its amylose content, a key determinant of texture, where a higher amylose content generally correlates with firmer and harder eating quality [31,32]. As shown in Figure 2c, the amylose content first exhibited a fluctuating upward trend, reaching a peak after 150 d of storage, followed by a gradual decline. At 180 d of storage, the amylose content under NCA, LT and LT + NCA treatments was 17.33%, 16.81% and 1 6.29%, respectively, all significantly lower than that in the CS group (18.32%, *p* < 0.05). These results clearly demonstrate that both NCA and LT storage conditions effectively mitigate the increase in amylose content of rice during storage. Furthermore, LT treatment showed a stronger effect than NCA alone, while the combined LT + NCA treatment yielded the most pronounced reduction. This aligns with the established understanding that higher storage temperature and humidity, along with longer storage time, can significantly alter amylose content [33]. Consequently, our findings indicate that LT storage, particularly in combination with NCA, effectively slows these undesirable changes, thereby better preserving the edible quality of rice.

#### 3.2.3. Pasting Properties

The pasting properties of rice starch serve as sensitive indicators of aging during storage [2]. Table 1 presents the effects of different storage conditions on pasting properties.

Peak viscosity (PV) reflects the swelling power of starch granules. PV first increases and then decreases during storage, a trend consistent with the change in amylose content (Figure 2c). After 180 d of storage, PV was significantly lower in the LT + NCA group than in the CS (*p* < 0.05). This might be linked to the increase in amylose content during storage, which can enhance the resistance of starch granules to rupture, thereby affecting starch gelatinization [34].

Final viscosity (FV) under CS conditions increased throughout storage, while FV under LT, NCA and LT + NCA showed a fluctuating upward trend. After 180 d of storage, FV was significantly lower in LT (2555.00 Pa s), NCA (2953.00 Pa s) and LT + NCA (2515.50 Pa s) compared with CS (2993.50 Pa s) (*p* < 0.05). These results indicate that LT, NCA and LT + NCA treatments can effectively delay the increase in final viscosity and improve the extensibility of rice starch gel. Notably, the LT + NCA combination maintained a more stable FV than LT alone, suggesting that nitrogen-modified atmosphere conditioning enhances the stabilizing effect of low-temperature storage, leading to more consistent gel extensibility and effectively extending the storage period of rice.

Breakdown viscosity (BD) reflects the stability of the starch hot paste, with a higher BD generally indicating greater starch granule rupture and better rice eating quality [2]. BD initially increased and subsequently decreased during storage. The early increase indicates that starch granules became more susceptible to rupture during cooking after storage. However, the later decrease suggests a significant reduction in the swelling and rupture capacity of starch granules, corresponding to a deterioration in edible and cooking quality in the later storage stage [33,34]. After 180 d of storage, BD was significantly higher in LT (974.00 Pa s) and LT + NCA (970.50 Pa s) than in CS (732.50 Pa s, *p* < 0.05), indicating better eating and cooking quality, which is consistent with the findings of Gu Li et al. (2025) [19].

Setback (SB) mainly results from the rearrangement of starch molecules in the system during cooling [2]. SB viscosity increased slowly as storage time increased. After the 180 d of storage, SB was significantly lower in LT (1235.00 Pa s) and LT + NCA (1214.50 Pa s) than in CS (1398.00 Pa s, *p* < 0.05). A lower SB indicates slower retrogradation and better maintenance of initial cooking quality, likely due to the mitigated degradation of amylose, which is in accordance with the results obtained above.

In conclusion, LT + NCA treatment slowed the decline in peak viscosity and breakdown while curbing the increase in final viscosity and setback, thereby preserving the cooking and eating quality of rice.

### 3.3. The Mycotoxins Content of Rice During Storage

The contamination of rice with fungal mycotoxins is a significant global concern, as it compromises grain safety, quality and nutritional properties. Key mycotoxins detected in rice include highly toxic aflatoxins (particularly AFB1 and OTA) [35,36,37].

**Figure 3:**
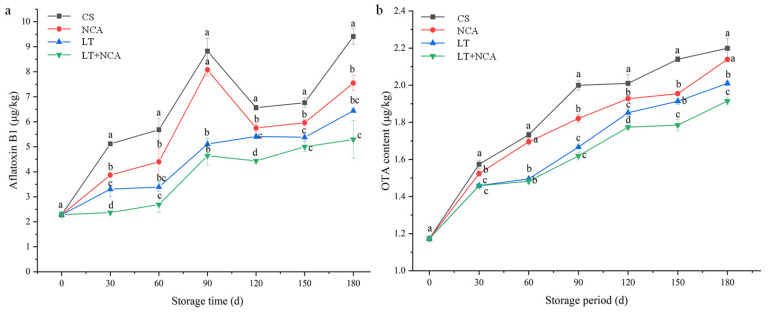
The AFB1 (**a**) and QTA (**b**) content of rice during storage. CS, conventional storage; LT, low temperature; NCA, nitrogen-controlled atmospheric conditions, LT + NCA, low temperature combined with nitrogen-controlled atmospheric conditions. Results with different letters (a–c) in the same storage period are significantly different (*p* < 0.05).

As shown in Figure 3, the contents of AFB1 and QTA in rice gradually increased with prolonged storage under CS, NCA, LT and LT + NCA conditions. The results indicated that extended storage duration promoted the accumulation of OTA and AFB1. After 180 d of storage, the lowest OTA content was observed under LT + NCA, followed by LT and NCA treatments. Chuaysrinule et al. (2024) [38] identified temperatures between 22 and 32 °C as posing the highest risk for OTA production. Qiu et al. (2024) [35] indicated that lower temperatures effectively inhibit microbial growth and toxin synthesis, whereas higher temperatures accelerate the accumulation of AFB1 and OTA. Notably, the contents of AFB1 and OTA in all treatment groups remained within established safety limits after 180 d of storage (Figure 3), which is consistent with the observation reported by T. Wang et al. (2024) [39]. This indicates that although mycotoxin content increases over time, appropriate storage conditions such as LT + NCA can effectively restrain this increase to within safe thresholds. It should be noted that the above inference of suppressed fungal proliferation is only indirectly supported by mycotoxin quantification data. Future work will quantify fungal colony counts and characterize fungal communities to provide direct microbial evidence supporting the present mycotoxin results.

### 3.4. Overview of Metabolites During Storage of Rice

Based on the above results, LT + NCA effectively delayed rice aging and prevented quality deterioration. To further investigate the regulatory mechanism by which LT + NCA storage affects rice preservation, metabolomics analysis was performed to profile the differential compounds among the sample groups. The extracted ion chromatogram (EIC) of the quality control (QC) samples collected at ESI+ and ESI- are shown in Figure S1. The retention time and response intensity stability of all QC samples are excellent, indicating good stability in the data acquisition of the instrument. The correlation coefficients for QC sample replicates in both modes exceeded 0.9, which signified minimal experimental errors and excellent data quality (Figure S2).

**Figure 4:**
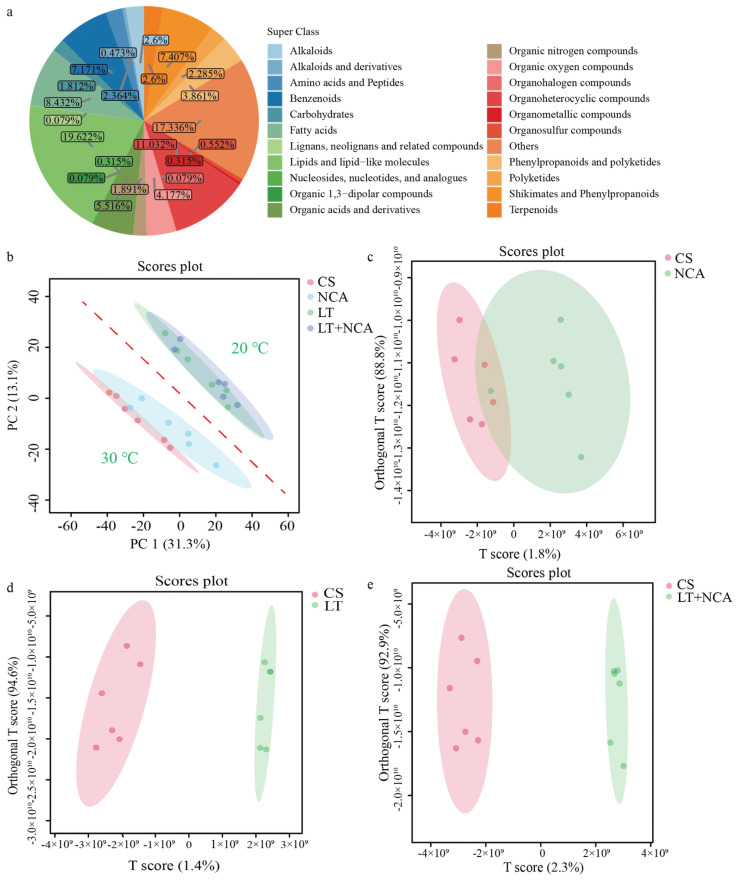
Multivariate statistical analysis of the metabolites present in rice under different storage treatments. Classification of the identified metabolites of rice (**a**). Principal component analysis of metabolic profiles of all samples (**b**). OPLS-DA analysis of metabolic profiles of CS vs. LT (**c**), CS vs. NCA (**d**) and CS vs. LT + NCA (**e**). CS, conventional storage; LT, low temperature; NCA, nitrogen-controlled atmospheric conditions, LT + NCA, low temperature combined with nitrogen-controlled atmospheric conditions.

Metabolomic analysis of rice identified 653 metabolites across the four groups under positive and negative ionization modes. As shown in Figure 4a, these metabolites were classified into several categories, including carbohydrates, fatty acids and conjugates, lipids and lipid-like molecules, organic acids and derivatives, organoheterocyclic compounds, amino acids and peptides, alkaloids and their derivatives, benzenoids, shikimates and phenylpropanoids, etc. Based on the 653 metabolites obtained in this study, PCA and OPLS-DA was performed to evaluate overall metabolic differences among the CS, NCA, LT and LT + NCA groups. As shown in Figure 4b–e, the dispersion of rice samples across different storage conditions was good, suggesting significant differences in the metabolite profiles among the storage conditions. Principal component 1 (PC1) and PC2 explained 31.3% and 13.1% of the total variance, respectively. In addition, the CS and NCA group were positioned closer to each other, while the LT and LT + NCA groups were also closely located, indicating that the metabolic profiles of rice were primarily separated by temperature. Permutation testing results indicated that the model was reliable, and had not experienced overfitting (Figure S3).

### 3.5. Screening for Differential Metabolites

**Figure 5:**
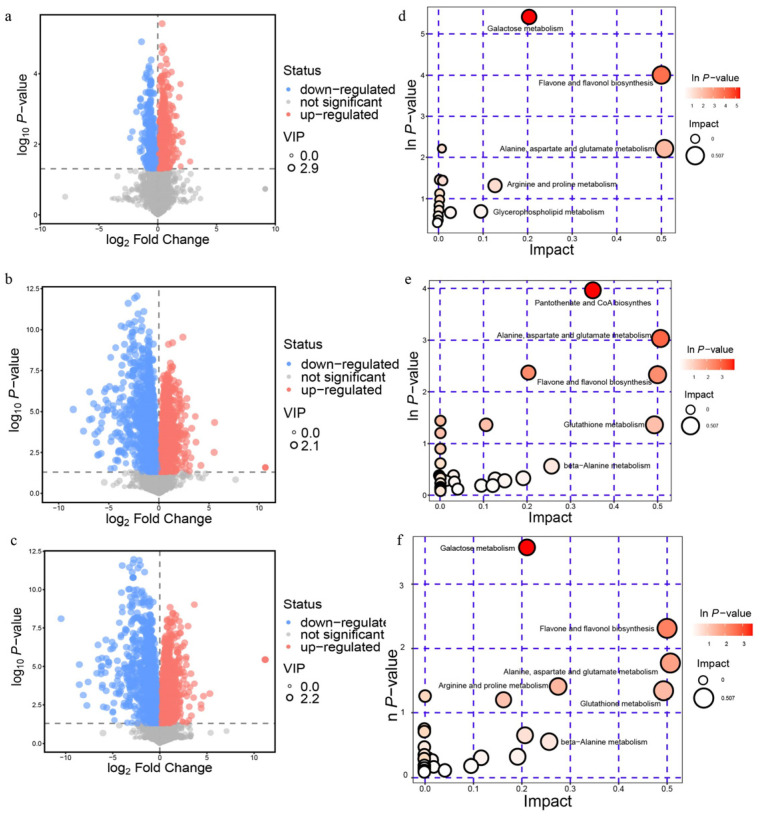
Volcano plot of the differential metabolites of CS vs. LT (**a**), CS vs. NCA (**b**) and CS vs. LT + NCA (**c**). Bubble diagram of metabolites pathways involved in differential rice metabolites of of CS vs. LT (**d**), CS vs. NCA (**e**) and CS vs. LT + NCA (**f**). CS, conventional storage; LT, low temperature; NCA, nitrogen-controlled atmospheric conditions, LT + NCA, low temperature with nitrogen-controlled atmospheric conditions.

To comprehensively understand the significant differences in metabolite profiles among CS, NCA, LT and LT + NCA samples, differential accumulation of metabolites was determined based on OPLS-DA models for the comparisons of CS vs. NCA, CS vs. LT and CS vs. LT + NCA. In this study, a threshold of VIP > 1 and false discovery ratio (FDR) < 0.05 was adopted to identify the differential metabolites that significantly contributed to the group separation, facilitating a deeper understanding of the characteristic products of different metabolic pathways under various storage conditions. Volcano plots (Figure 5a–c) further visualize these differential metabolites, where each point represents a metabolite and point size corresponds to its VIP value from the OPLS-DA model.

### 3.6. Analysis of Metabolic Pathways

**Figure 6:**
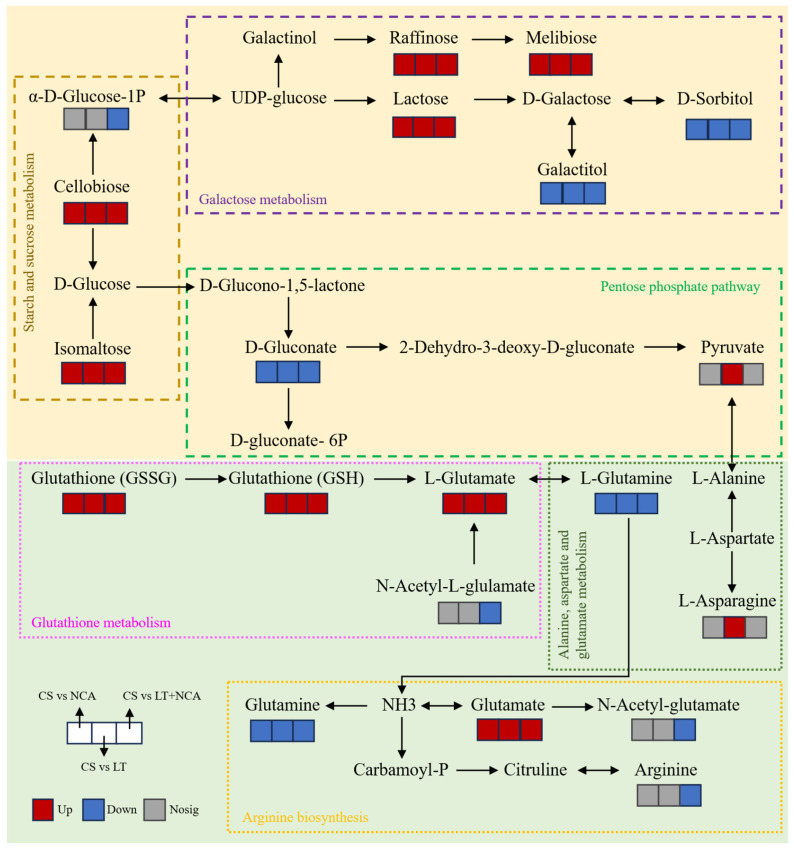
Metabolites interrelationship map. The red font indicates a higher expression of the substance than CS, while the blue font denotes a lower expression of the substance than CS. The black font is used to represent no change between the two groups.

In organisms, various metabolites coordinate to form complex metabolic pathways, thereby collectively completing biological functions. To further analyze the relationships among differential metabolites, Kyoto Encyclopedia of Genes and Genomes (KEGG) enrichment analysis was employed to identify the most relevant metabolic pathways. As shown in Figure 5, each bubble represents an identified metabolic pathway. The most significantly enriched KEGG pathways among the differential metabolites identified in CS vs. NCA samples included alanine, aspartate and glutamate metabolism, flavone and flavonol biosynthesis, galactose metabolism (Figure 5d). For CS vs. LT samples, the most significantly enriched KEGG pathways included alanine, aspartate and glutamate metabolism, flavone and flavonol biosynthesis, glutathione metabolism, pantothenate and CoA biosynthesis and beta-alanine metabolism (Figure 5e). The metabolic pathways of alanine, aspartate and glutamate metabolism, flavone and flavonol biosynthesis, glutathione metabolism, arginine and proline metabolism, beta-alanine metabolism and galactose metabolism were identified as key pathways for maintaining the storage quality of rice under LT + NCA treatment (Figure 5f). From the above analysis, it is evident that there were significant metabolic differences between rice stored under CS and LT + NCA conditions. To further highlight the relationships of metabolites involved in rice metabolism, Figure 6 summarizes the connections among key metabolites and metabolic pathways.

#### 3.6.1. Carbohydrate Metabolism

Carbohydrate metabolism is the primary source of energy and carbon skeletons for rice grains, and its dynamic regulation is closely related to starch structure stability, cooking quality, and stress tolerance during storage [40]. The main involved metabolic pathways included galactose metabolism, starch and sucrose metabolism and pentose phosphate pathway. The key differential metabolites included lactose, raffinose, melibiose, D-sorbitol, galactitol, cellobiose, α-D-Glucose-1P, isomaltose, D-gluconate, pyruvate.

During galactose metabolism, LT and NCA treatments increased the contents of lactose, raffinose and melibiose, while reducing the contents of galactitol and D-sorbitol. Raffinose family oligosaccharides are classic compatible solutes in cereal grains, which can stabilize cell membrane and protein structures, scavenge ROS, and enhance abiotic stress tolerance. As a type of carbohydrate in plants, raffinose may alleviate the effects of salt stress by regulating cellular osmotic pressure. In summary, raffinose is an important trisaccharide that plays a crucial role in galactose metabolism in plants [40]. Morsy et al. (2007) [41] reported that raffinose may increase chilling tolerance through its role in membrane stabilization via interaction with phospholipid headgroups. In contrast, sugar alcohol synthesis is a relatively energy-consuming osmotic regulation pathway, and its inhibition reduces unnecessary energy consumption. The preferential accumulation of functional oligosaccharides mediated by LT + NCA treatment effectively enhances cell membrane stability, which is consistent with the observed reductions in MDA content and electrolyte leakage, thereby further delaying rice quality deterioration.

Starch and sucrose are two important forms of energy storage in plants [40]. Starch and sucrose metabolism has important biological significance in plant physiology and plays a crucial role in plant growth, development and environmental adaptation. This core energy metabolism pathway in rice grains was significantly modulated by LT + NCA treatment. The contents of cellobiose and isomaltose, key hydrolysis products of starch and sucrose, were significantly upregulated in the LT + NCA group, indicating moderate enhancement of starch and sucrose hydrolysis to maintain basic energy supply for cellular metabolism under hypothermic and hypoxic conditions. Meanwhile, α-D-glucose-1-phosphate showed differential accumulation, suggesting a redirection of glucose metabolic flux away from conventional glycolysis toward other stress-resistant metabolic branches. Moderate starch hydrolysis avoids excessive starch degradation and structural damage, which is consistent with the observed better maintenance of pasting properties (higher breakdown viscosity, lower final viscosity and setback) in this study. A higher level of soluble sugars is associated with ROS scavenging [42].

The pentose phosphate pathway (PPP), a major pathway for oxidative glucose decomposition and NADPH production [43], was significantly inhibited by LT + NCA treatment, as evidenced by the significant downregulation of D-gluconate, a key intermediate of the PPP oxidative branch. Excessive PPP activity accelerates ROS production and oxidative damage in stored grains, and its inhibition is an important adaptive strategy to reduce oxidative stress under hypothermic and hypoxic conditions [15]. Pyruvate, a key intermediate connecting carbohydrate and amino acid metabolism, showed condition-specific changes, indicating finely tuned crosstalk between carbohydrate and amino acid networks. The coordinated inhibition of PPP and redirection of carbohydrate metabolic flux reduced oxidative damage, stabilized grain microstructure, and synergistically contributed to the superior quality retention of rice under LT + NCA storage. Furthermore, PPP activation supplies NADPH to fuel the glutathione reductase reaction, enabling the conversion of GSSG back to GSH and maintaining cellular redox balance. This metabolic coordination between PPP and glutathione metabolism creates an integrated antioxidant system that counteracts ROS accumulation during rice aging.

#### 3.6.2. Amino Acids Metabolism

Amino acids are unique taste compounds in cooked rice and serve as the primary substrates for protein synthesis and various metabolic reactions. Amino acid metabolites may play a pivotal role in the quality formation of premium rice varieties [44]. Amino acid metabolism is a core regulatory hub for postharvest cereal stress resistance, redox homeostasis, and nutrient retention, as amino acids act as critical nitrogen carriers, protein building blocks, and precursors of antioxidant and stress-responsive metabolites. Disruptions in amino acid metabolic homeostasis directly accelerate lipid peroxidation, membrane damage, and quality loss in stored rice. In this study, LT and NCA treatments significantly modulated multiple critical amino acid metabolic pathways, mainly involving glutathione metabolism; alanine, aspartate and glutamate metabolism; arginine biosynthesis; and D-amino acid metabolism. The key differential metabolites included glutathione disulfide (GSSG), glutathione (GSH), glutamate, arginine, glutamine, L-glutamate, L-glutamine, L-asparagine and N-acetyl-L-glulamate.

GSH metabolism constitutes the most direct line of defense against oxidative damage in stored grains. GSH, a tripeptide synthesized from glutamate, cysteine, and glycine, functions as the primary low-molecular-weight antioxidant that maintains cellular redox homeostasis and scavenges reactive oxygen species (ROS) generated during grain aging. The GSH pathway serves as a central hub for redox regulation and antioxidant defense in plants. During rice storage, GSH maintains redox balance by cycling between its reduced (GSH) and oxidized (GSSG) forms. Glutamine–glutamate interconversion is the central axis of nitrogen transport and carbon skeleton redistribution in cereal grains, regulating the allocation of nitrogen resources for diverse physiological processes. Compared with the control group, LT + NCA treatment induced a significant decrease in L-glutamine content and a remarkable increase in L-glutamate content in rice grains, indicating accelerated catalytic conversion of glutamine to glutamate. Glutamate, as a key precursor for multiple defense metabolites, provides sufficient substrate support for downstream glutathione synthesis, avoiding excessive nitrogen consumption for redundant anabolic processes and ensuring efficient utilization of limited nutrients during long-term storage.

Arginine biosynthesis was significantly inhibited under LT + NCA storage conditions, as reflected by the downregulation of N-acetyl-L-glutamate and arginine content in rice grains. Arginine synthesis is an energy-intensive anabolic process associated with grain growth and development, which is not essential for postharvest stress resistance. The inhibition of arginine biosynthesis in this study allowed more nitrogen resources derived from glutamate to be redirected to glutathione synthesis, rather than being used for unnecessary growth-related metabolism. This strategic metabolic rearrangement enhances the adaptive capacity of rice grains to storage stress and contributes to long-term quality retention.

### 3.7. Correlation Analysis Between Key Metabolites and Physicochemical Quality Parameters

**Figure 7:**
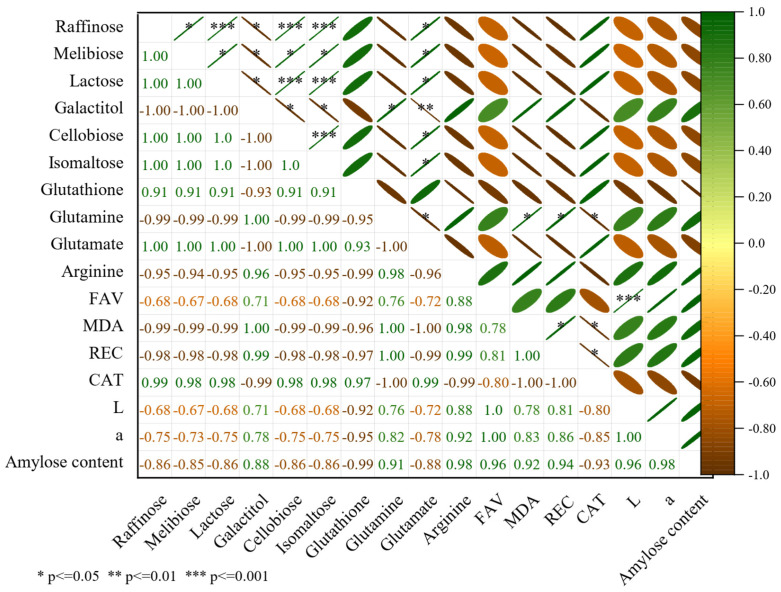
Correlation analysis between key metabolites and quality indices.

The correlation thermogram of FAV, MDA, REC, CAT, color, amylose content and ten key metabolites of rice samples during storage was displayed in Figure 7. Noticeably, the FAV and MDA exhibited a significant negative correlation with raffinose, melibiose, lactose, cellobiose, isomaltose, glutathione, glutamate, and a positive correlation with galactitol, glutamine and arginine, respectively. Conversely, CAT showed positive a correlation with raffinose, melibiose, lactose, cellobiose, isomaltose, glutathione, glutamate, and a negative correlation with galactitol, glutamine and arginine, respectively. These results were consistent with rice quality and the expression level changes of key metabolites (Figure 1 and Figure 6). Although untargeted metabolomics screened candidate metabolites linked to rice preservation, this study has an evident limitation: these key metabolites were not validated via targeted quantification. In future work, targeted metabolomics will be performed to resolve this issue.

## 4. Conclusions

In summary, the combined application of LT + NCA offers a synergistic preservation effect, outperforming either treatment alone. LT + NCA storage effectively suppresses lipid peroxidation, mitigates cell membrane damage, and significantly reduces the increase in FAV, MDA content and REC during rice storage, thereby better maintaining storage quality. In addition, LT + NCA treatment slowed the decline in peak viscosity and breakdown while curbing the increase in final viscosity and setback, resulting in preserving the cooking and eating quality of rice. Furthermore, LT + NCA storage resulting in the lowest accumulation of AFB1 and OTA, thereby extending the safe storage window. Finally, untargeted metabolomics analysis further demonstrated that LT + NCA treatment increased the levels of antioxidant metabolites and inhibited the pentose phosphate pathway in rice, thereby boosting the antioxidant defense system. In conclusion, LT + NCA treatment can serve as a promising nonthermal processing technique to enhance the storage stability of rice.

## Figures and Tables

**Table 1:** Pasting property changes of rice during storage.

Storage Period (d)	Treatment	Gelatinization Characteristics				
		Peak Viscosity (Pa s)	Final Viscosity (Pa s)	Breakdown (Pa s)	Setback (Pa s)	Pasting Temperature (°C)
0	Fresh	1995.00 ± 31.11	1173.50 ± 2.12	821.50 ± 28.99	1105.00 ± 11.31	88.35 ± 0.00
30	CS	2466.00 ± 5.66 a	2577.00 ± 14.14 a	1098.50 ± 20.51 a	1209.50 ± 0.71 a	83.10 ± 0.57 c
	LT	2271.00 ± 16.97 bc	2402.00 ± 5.66 b	1017.00 ± 15.56 b	1148.00 ± 4.24 b	85.90 ± 0.00 b
	NCA	2340.50 ± 38.89 b	2557.00 ± 48.08 a	1007.50 ± 9.19 b	1224.00 ± 18.38 a	85.93 ± 0.04 b
	LT + NCA	2229.50 ± 26.16 c	2406.50 ± 26.16 b	987.50 ± 12.02 b	1164.50 ± 12.02 b	87.08 ± 0.60 a
60	CS	2443.00 ± 35.36 b	2638 ± 28.28 ab	1000.50 ± 12.02 b	1195.5 ± 19.09 ab	84.38 ± 0.11 a
	LT	2546.00 ± 60.81 ab	2491.00 ± 28.28 c	1189.00 ± 41.01 a	1134.00 ± 8.49 b	79.50 ± 0.00 c
	NCA	2630.00 ± 15.56 a	2697.50 ± 3.54 a	1178.00 ± 8.49 a	1245.50 ± 3.54 a	80.30 ± 0.07 c
	LT + NCA	2481.50 ± 28.99 b	2503.00 ± 89.1 bc	1141.50 ± 0.71 a	1163 ± 60.81 ab	81.50 ± 0.57 b
90	CS	2374.00 ± 26.87 a	2778.00 ± 32.53 a	896.00 ± 1.41 b	1300.00 ± 7.07 a	85.53 ± 0.6 b
	LT	2293.50 ± 28.99 a	2438.00 ± 29.70 c	982.00 ± 8.49 a	1126.50 ± 9.19 c	86.30 ± 0.64 ab
	NCA	2106.00 ± 60.81 b	2650.00 ± 56.57 b	709.50 ± 30.41 c	1253.50 ± 26.16 b	87.10 ± 0.57 a
	LT + NCA	2182.50 ± 24.75 b	2499.00 ± 11.31 c	849.00 ± 32.53 b	1165.50 ± 3.54 c	87.58 ± 0.04 a
120	CS	2246.50 ± 28.99 a	2812.50 ± 4.95 a	747.50 ± 27.58 c	1313.5 ± 3.54 a	86.78 ± 0.04 ab
	LT	2264.50 ± 2.12 a	2455.50 ± 10.61 c	951.00 ± 29.7 a	1142 ± 16.97 b	86.33 ± 0.6 bc
	NCA	2251.50 ± 4.95 a	2769.50 ± 0.71 b	788.50 ± 3.54 c	1306.5 ± 2.12 a	85.90 ± 0.07 c
	LT + NCA	2100.00 ± 4.24 b	2355.00 ± 16.97 d	861.00 ± 15.56 b	1116.00 ± 2.83 c	87.50 ± 0.00 a
150	CS	2284.50 ± 37.48 a	2971.50 ± 24.75 a	723.50 ± 13.44 c	1410.50 ± 0.71 a	87.50 ± 0.07 a
	LT	2287.00 ± 38.18 a	2509.00 ± 24.04 b	1004.50 ± 34.65 a	1226.50 ± 20.51 b	85.93 ± 1.17 a
	NCA	2396.00 ± 4.24 a	2936.00 ± 7.07 a	893.00 ± 1.41 b	1433.00 ± 4.24 a	85.93 ± 0.04 a
	LT + NCA	2337.50 ± 62.93 a	2555.00 ± 42.43 b	1031.00 ± 26.87 a	1248.50 ± 6.36 b	86.33 ± 0.53 a
180	CS	2328.00 ± 5.66 a	2993.50 ± 3.54 a	732.50 ± 3.54 b	1398.00 ± 12.73 b	86.75 ± 0.00 b
	LT	2294.00 ± 25.46 ab	2555.00 ± 7.07 c	974.00 ± 15.56 a	1235.00 ± 2.83 c	85.83 ± 0.04 c
	NCA	2183.00 ± 1.41 c	2953.00 ± 14.14 b	661.50 ± 2.12 c	1431.50 ± 10.61 a	87.55 ± 0.07 a
	LT + NCA	2271.50 ± 17.68 b	2515.50 ± 0.71 d	970.50 ± 20.51 a	1214.50 ± 3.54 c	86.73 ± 0.04 b

Note: Within the same storage time, values in each column with different letters indicate significant differences at *p* < 0.05. CS, conventional storage, LT, low temperature, NCA, nitrogen-controlled atmospheric conditions, LT + NCA, low temperature combined with nitrogen-controlled atmospheric conditions.

## Data Availability

The raw data supporting the conclusions of this article will be made available by the authors on request.

## Supplementary Materials

The following supporting information can be downloaded at: https://www.mdpi.com/article/10.3390/foods15132326/s1, Figure S1: The extracted ion chromatogram (EIC) of the internal standard positive (a) and negative (b) ions for all QC samples; Figure S2: Correlation analysis of QC samples; Figure S3: Permutation test of OPLS-DA model.
