# Supplementary material for: Metabolomics Analysis Unveils the Underlying Mechanism of Low-Temperature Combined with Nitrogen Modified Atmosphere in Delaying Quality Deterioration of Rice
Source: Foods. 2026 Jul 1;15(13):2326. doi: 10.3390/foods15132326 (PMC13361427; doi:10.3390/foods15132326)
Supplement: Supplementary file 1 [file foods-15-02326-s001.zip › foods-4370205-supplementary.pdf]

a

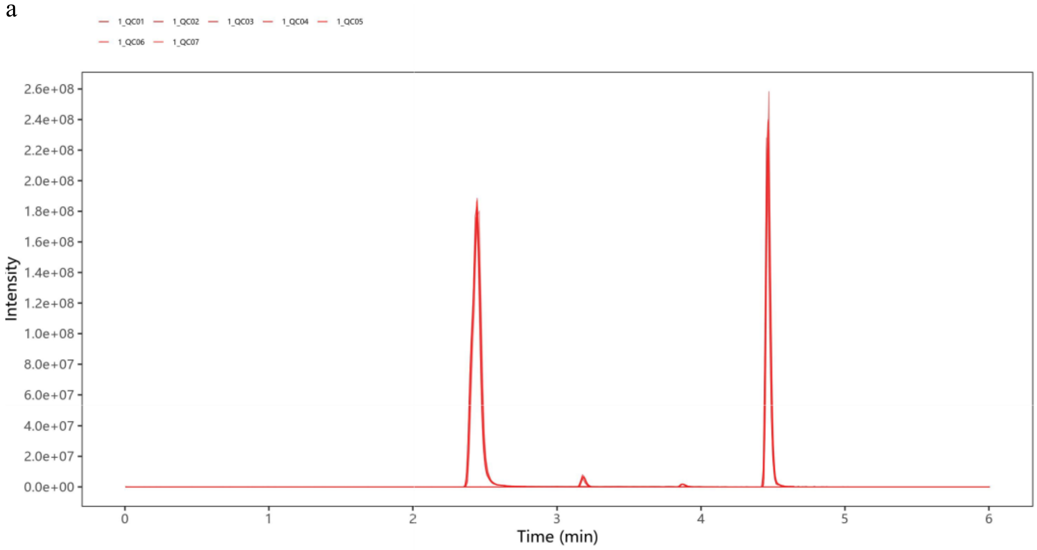

b

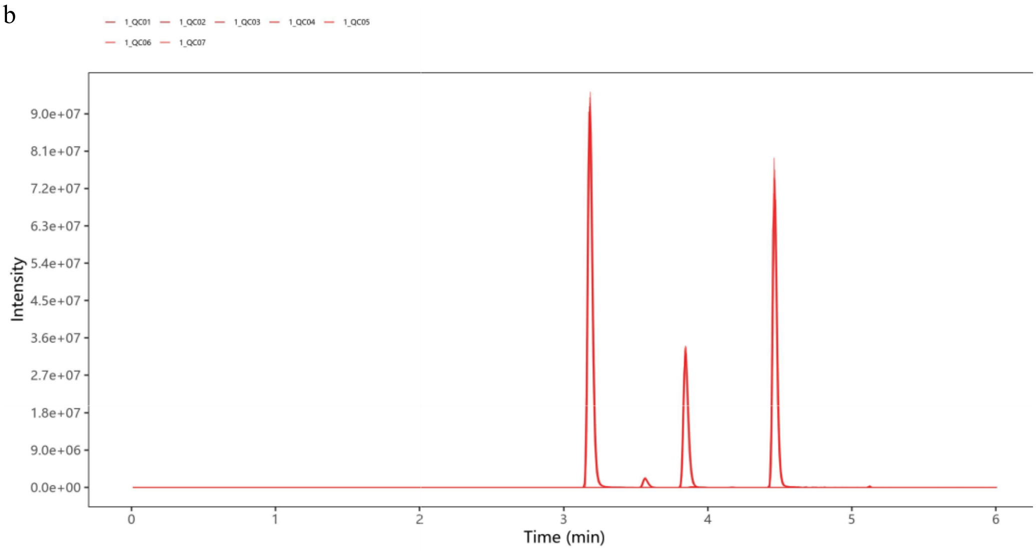

Figure S1 The extracted ion chromatogram (EIC) of the internal standard positive (a) and negative (b) ions for all QC samples.

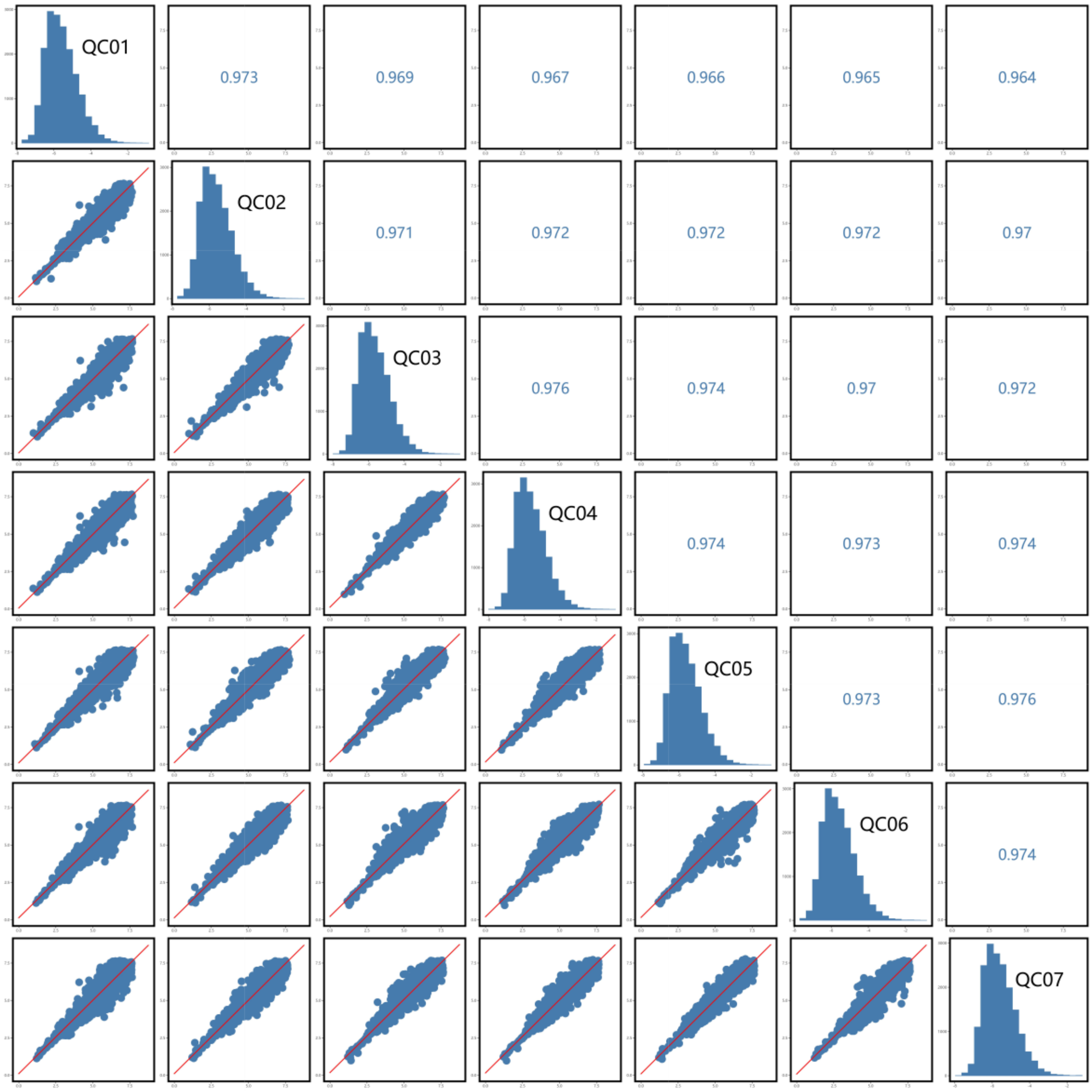

Figure S2 Correlation analysis of QC samples.

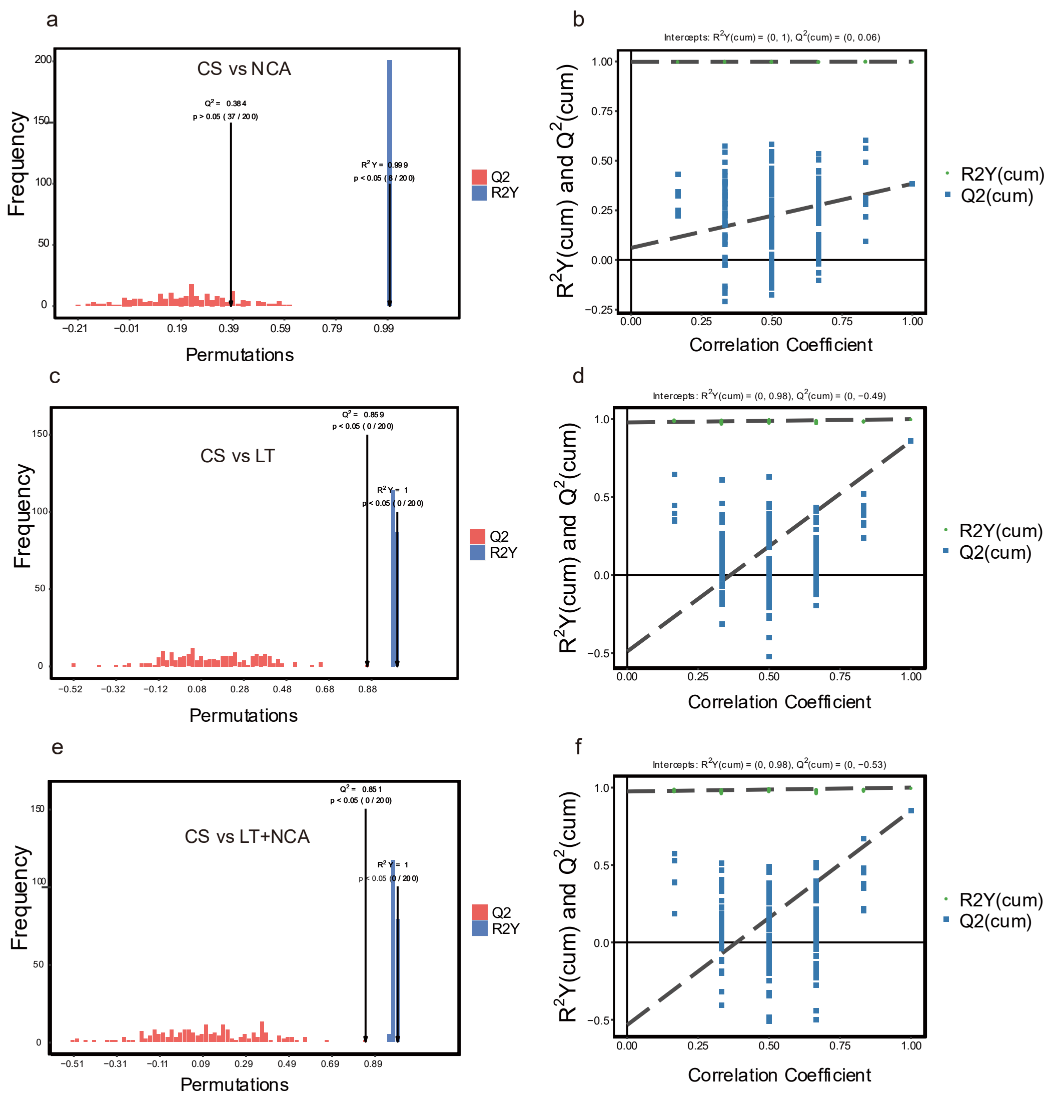

Figure S3 Permutation test of OPLS-DA model.
